# Comentario sobre el artículo “Metapresencialidad: concepto fundante de una teoría crítica de la salud digital” de Naomar de Almeida Filho

**DOI:** 10.18294/sc.2024.5069

**Published:** 2024-06-11

**Authors:** Oriol Alonso Cano, Eulàlia Hernández Encuentra

**Affiliations:** 1 Doctor en Filosofía. Profesor, Universitat Oberta de Catalunya, Barcelona, España. oalonsoca@uoc.edu Universitat Oberta de Catalunya Universitat Oberta de Catalunya Barcelona Spain oalonsoca@uoc.edu; 2 Doctora en Psicología. Profesora agregada, Universitat Oberta de Catalunya. Miembro, ePsicología del Col.legi de Psicòlegs de Catalunya, Barcelona, España. ehernandez@uoc.edu Universitat Oberta de Catalunya Universitat Oberta de Catalunya Barcelona Spain ehernandez@uoc.edu

Naomar de Almeida Filho inicia su propuesta^1^ abordando la temática de lo tecnológico, a través del trabajo de Álvaro Vieria Pinto^2^, quien propone una diferenciación entre lo que es tecnología, técnica y objeto tecnológico. Esta distinción es necesaria, según Almeida Filho, para apreciar las implicaciones ontológicas e ideológicas de cualquier identificación *tecnocentrista*. Así pues, técnica “se refiere a la forma en que se realizan los actos productivos del ser humano, materializándose en instrumentos, máquinas y artefactos que transforman la naturaleza, humanizándola a través de la cultura”^1^. La técnica es un hacer, una actividad humana, lo cual nos remite a célebres concepciones antropológicas como antropotécnica, tal y como muchas veces plantea, por ejemplo, Peter Sloterdijk^3^. El objeto tecnológico designa el producto de ese hacer, mientras que la tecnología aborda diferentes cuestiones (ciencia de la técnica; discurso sobre el conjunto de las técnicas; todo el conocimiento producido y acumulado históricamente e ideología de lo tecnológico). El objetivo de todo este proceso es salir del tecnocentrismo que presenta a un sujeto enajenado que no es capaz de percibir a la máquina como el producto de su trabajo. Sin embargo, pese a que la distinción entre técnica y objeto tecnológico parece evidente, el autor no acaba de ser específico en el momento de definir y cartografiar de manera precisa la noción de tecnología. La tecnología, ¿es un discurso?, ¿una ciencia?, ¿un conjunto de conocimientos?, ¿una ideología?, ¿una ontología?, ¿un híbrido de todas ellas? Es en este punto en el que la precisión que tanto pretende buscar el autor se diluye progresivamente.

A continuación, Naomar de Almeida Filho se dirige hacia la teoría de Milton Santos^4^^,^^5^ para abordar críticamente los conceptos, prácticas, estrategias y dispositivos de las tecnologías digitales. En particular, concibe el espacio como un conjunto inseparable de sistemas de objetos y acciones. El espacio es un constructo, una mezcla, conjunción que se va modificando históricamente, lo cual remite, por cierto, a concepciones materialistas y constructivistas. Asimismo, este espacio que muta, configura un entorno técnico-científico-informativo en el que producimos mundo y realidad a través, entre otros factores, de la técnica. La hibridación llega a ser tal que no se puede distinguir el entorno geográfico del técnico. Aquí, en esta cuestión, el autor parece no atender a cuestiones ideológicas del entorno. Dicho de otra manera, las construcciones, mutaciones, variaciones responden a unas dinámicas ideológico-políticas determinadas, así como a unos determinados intereses implícitos, tal y como podemos observar en los trabajos de Henri Lefebvre^6^, pero sobre todo de Manuel Delgado^7^, Saskia Sassen^8^ o David Harvey^9^. El autor menciona este hecho pero, al mismo tiempo, parece obliterarse en su abordaje.

Luego, en ese contexto, el autor aborda el concepto de tecnología digital que, particularmente:

“...se refiere a técnicas (procedimientos, protocolos, directrices) y objetos técnicos (equipos, dispositivos) cuya funcionalidad y operación efectiva dependen de programas y lenguajes (sistemas operativos, lenguaje de programación, algoritmos) habilitados por sistemas lógicos o secuencias de comandos formulados en códigos binarios”.^1^

Si esto es así, y volviendo a la idea inicial de escapar del tecnocentrismo, nos encontramos con un concepto que, pese a todas las tentativas de situar su génesis en el hacer humano, llega a alcanzar un nivel tal de complejidad y heterogeneidad, que acaba distanciándose del nivel de programación inicial humano. Es decir, si todo depende de programas y lenguajes habilitados por sistemas lógicos o secuencias de formulaciones binarias, ¿podemos seguir diciendo que la técnica es una actividad estrictamente humana?, ¿no caemos en los peligros de la alienación que tanto intentaba eludir el autor en su distinción entre técnica, objeto técnico y tecnología?, ¿no es volver a los peligros prometeicos de un sistema que, pese a tener su génesis humana, se desarrolla, dada su complejidad sistémica, con independencia de los lenguajes y sintaxis humana?

Posteriormente, el autor aborda la temática de la información a partir de la teoría del realismo informacional ontológico que, en resumidas cuentas, nos plantea la imposibilidad de una teoría unificada de la información, así como una definición de esta como una entidad “cuantificable, plausible, acumulativa, almacenable y transmisible”^1^, más allá de señalar la independencia de la información respecto al medio físico en el que circula, así como realizar un abordaje cognitivo-ontológico-ético en el que se nos define y articula la naturaleza de la información. Si nos vamos a la dimensión ética, se nos apunta que es necesaria una microética que identifique problemas emergentes y vaya a una prevención o a una corrección para abordar los efectos negativos de la información. Ahora bien, ¿qué sucede con la naturaleza misma de la información y los intereses que hay en ella? El autor se centra en los impactos de la información, en sus efectos subjetivos, sin embargo, parece olvidarse, por un lado, de los intereses que entran en juego en el flujo informativo y, por el otro, de la misma naturaleza ontológica de la información. Se olvida de cómo debemos leer esa información, de problematizarla más allá de asumirla con mayor o menor grado de pasividad. 

A su vez, la información son datos traducidos a un nivel cognitivo, pero toda traducción implica una transformación de lo traducido. Es decir, siempre hay una pérdida, cambio, traición cuando traducimos un discurso. Así pues, ¿los datos corresponden verdaderamente a la información?, ¿qué naturaleza tiene estos datos?, ¿no nos estaremos moviendo por un dualismo de reminiscencias platónicas o bien por una dimensión kantiana al distinguir una realidad fenoménica a diferencia de otra nouménica? Es decir, que el realismo informacional de Luciano Floridi^10^ es más que cuestionable en términos cognitivo-ontológico-epistemológicos. 

Sobre la cuestión de la metapresencia, el autor aborda una genealogía del término que remite a Marcus Alves^11^, aunque la génesis podríamos apreciarla en la teoría del simulacro de Jean Baudrillard^12^ o la *hauntología* de Jacques Derrida^13^. En este sentido, es muy interesante porque conecta plenamente con una deconstrucción de la noción de presencialidad y nos ubica en un espacio en el que realidad/ficción, ausencia/presencia parecen periclitarse por completo. Ahora bien, esto nos introduce hacia una deslocalización tanto identitaria como topológica. En esta tesitura, ¿dónde ubicar la responsabilidad de las acciones?, ¿hay un yo real separado del yo virtual al cual imputar la responsabilidad última de su quehacer? Y en un terreno más macro, ¿bajo qué marco jurídico-político situar determinados efectos metapresenciales?

Al abordar la idea que da título al artículo, para el origen de la definición de lo que hoy se conoce como salud digital, el autor escogió la tecnología, un aspecto material como concepto fundante. Sin embargo, en su contribución a una perspectiva de la salud digital crítica, elige la presencia, un aspecto subjetivo y experiencial. Es decir, adopta una doble postura crítica, por tanto. La definición propuesta por el autor, presenta la salud digital como un ecosistema complejo que era difícil vislumbrar en la primera aparición del término en 1995, e incluso en la definición “clásica” más conocida^14^. Aunque es posible que, en ese momento, no solo fuera un escenario inimaginable, sino también poco deseable, puesto que torpedeaba el santuario de la atención médica tradicional. Un imaginario que parece mantener de algún modo el autor, puesto que repetidamente alude a la atención “médica”, reduciendo excesivamente el alcance de la intervención en el campo de la salud. En cualquier caso, definir distintas formas de presencia (el autor las define como formas de metapresencia), son incuestionables hoy en día en la intervención en el campo de la salud.

Más allá de la metapresencialidad, los autores de este comentario proponen la consideración de los datos (*data*) como concepto fundante de ambas, la salud digital y la salud digital crítica actuales. Los datos han cambiado no solo la atención sanitaria (a nivel individual, colectiva y poblacional, como señala el autor) sino también la consideración del cuerpo, la salud y la enfermedad misma. Pero este punto, en todo caso, sería objeto de otra discusión.

Finalmente, queremos señalar el paralelismo que el autor establece entre las instituciones académicas y sanitarias respecto al tema de la metapresencialidad. Tal como establece el autor, que estuvo al frente de la Universidad Federal del Sur de Bahía, los autores firmantes de este comentario son profesores de la Universitat Oberta de Catalunya (Barcelona, España), la primera universidad nacida *en* Internet, es decir, que desarrollaba y desarrolla (desde 1995) toda su actividad académica, de investigación y de gestión, íntegramente en línea. En su creación, el rector fundador visionaba una universidad que rompiera las barreras espaciotemporales, y efectivamente erigió una universidad con mandato público sin distancias, por oposición a las *universidades a distancia* existentes en aquel momento.
